# Estrogen Receptor Signaling and the PI3K/Akt Pathway Are Involved in Betulinic Acid-Induced eNOS Activation

**DOI:** 10.3390/molecules21080973

**Published:** 2016-07-25

**Authors:** Nicolas Hohmann, Ning Xia, Katja Steinkamp-Fenske, Ulrich Förstermann, Huige Li

**Affiliations:** 1 Department of Pharmacology, Johannes Gutenberg University Medical Center, Obere Zahlbacher Straße 67, Mainz 55131, Germany; Nicolas.Hohmann@med.uni-heidelberg.de (N.H.); xianing@uni-mainz.de (N.X.); Katja.Steinkamp@gmx.de (K.S.-F.); Ulrich.Forstermann@Uni-Mainz.de (U.F.); 2 Department of Clinical Pharmacology and Pharmacoepidemiology, University of Heidelberg, Im Neuenheimer Feld 410, Heidelberg 69120, Germany

**Keywords:** betulinic acid, endothelial nitric oxide synthase, endothelial cells, estrogen receptor, PI3K, Akt

## Abstract

Betulinic acid (BA) is a naturally occurring pentacyclic triterpenoid with anti-inflammatory, antiviral and anti-cancer properties. Beneficial cardiovascular effects such as increased nitric oxide (NO) production through enhancement of endothelial NO synthase (eNOS) activity and upregulation of eNOS expression have been demonstrated for this compound. In the present study, immortalized human EA.hy 926 endothelial cells were incubated for up to 1 h with 1–100 µM BA and with the phosphatidylinositol-3-kinase (PI3K) inhibitors LY294002 and wortmannin, or the estrogen receptor (ER) antagonist ICI 182,780. Phosphorylation status of eNOS and total eNOS protein were analyzed by Western blotting using a serine 1177 phosphosite-specific antibody. Bioactive NO production was assessed by determination of cGMP content in rat lung fibroblasts (RFL-6) reporter cells. Short-term incubation of EA.hy 926 cells with BA resulted in eNOS phosphorylation at the serine 1177 residue in a concentration- and time-dependent manner with a half-maximal effective concentration of 0.57 µM. This was associated with an enhanced production of NO. BA-induced eNOS phosphorylation and NO production was completely blocked by pretreatment with ICI 182,780, and was attenuated by pretreatment with the PI3K inhibitors wortmannin and LY294002. These results indicate that fast non-genomic effects of ER with downstream signaling through the PI3K/Akt pathway and consecutive eNOS phosphorylation at serine 1177 are involved in BA-induced eNOS activation.

## 1. Introduction

Nitric Oxide (NO) generated by the endothelial NO synthase (eNOS) localized in vascular endothelial cells is a key player in cardiovascular health [1]. NO leads to vascular smooth muscle cell relaxation by activating the soluble guanylyl cyclase [2,3] and hence contributes to blood pressure control. Pharmacological inhibition of NO synthesis causes peripheral vasoconstriction and elevation of blood pressure [4]. The counter-regulation of NO-mediated peripheral vasodilatation accounts for 70% of the basal noradrenalin release in rabbits [5]. Mice with a deleted eNOS gene lack endothelium-dependent NO-mediated vasodilation and are hypertensive [6]. Beside its antihypertensive properties, NO propagates multiple anti-atherosclerotic effects [1]. It prevents leukocyte adhesion to the vascular endothelium and migration into the vascular wall [7]. NO reduces the endothelial permeability and thus the influx of lipoproteins into the vascular wall and inhibits oxidation of low-density lipoprotein. Furthermore, NO inhibits proliferation of vascular smooth muscle cells. Pharmacological blockade of eNOS leads to an accelerated rate of atherosclerosis formation [8,9]. NO also protects blood vessels from thrombosis by inhibiting platelet aggregation and adhesion. Endothelial NO release towards the vascular lumen is a potent inhibitor of platelet aggregation and adhesion to the vascular wall, preventing thrombosis and the release of platelet derived growth factor [10].

Reduced NO synthesis or increased NO degradation can be demonstrated in pathological conditions associated with cardiovascular and cerebrovascular diseases such as hypercholesterolemia, diabetes mellitus, hypertension, smoking and cerebral vascular stroke. Because of the antithrombotic, anti-atherosclerotic, and antihypertensive properties of bioactive endothelial NO, the vascular NO system is an attractive drug target for the prevention or therapy of cardiovascular disease [11]. Control of vascular NO synthesis is multilayered and occurs through transcriptional regulation mechanisms, posttranslational modification mechanisms, localization of eNOS protein, and direct interaction with its binding partners [12]. Post-translational modification involves acylation, acetylation, nitrosylation, glucosylation, glutathonylation and most importantly, phosphorylation. Changes in phosphorylation state allow rapid integrative response to different mechanical, humoral, metabolic, or pharmacological stimuli [12]. Multiple serine (Ser), threonine (Thr) and tyrosine residues with regulatory potential have been described. Major changes in enzyme function have been reported for the phosphorylation of Ser^1177^ (activation) and Thr^495^ (inhibition) in the human eNOS sequence. These mechanisms together with eNOS expression levels and eNOS functionality, are determining factors of vascular NO production [13]. The phosphorylation state of the Ser^1177^ residue is under control of a number of distinct kinases, such as Akt, protein kinase A, AMP-activated protein kinase (AMPK), calcium/calmodulin-dependent kinase (CaMK) II, and check point kinase I. Further upstream, Akt is under control of phosphatidyl-inositol-3-kinase (PI3K) and integrates signals from a number of positive effectors of eNOS activity such as vascular endothelial growth factor, insulin, and notably estrogen [14,15].

A clinically relevant cardiovascular-protective effect of estrogen has been demonstrated based on the lower incidence of coronary heart disease in pre-menopausal women relative to that of age-matched males. This protective effect is lost in post-menopausal women [16]. The estrogen receptor (ER) is a class I member of the nuclear receptor superfamily, defined as a ligand-activated transcription factor [17]. Three distinct isoforms are currently known: ERα, ERβ [18,19,20], and the G protein-coupled receptor GPR30 [21]. The full length human ERα is a 66 kDa protein with a length of 595 amino acids. The full length human ERβ is smaller, having a total length of 530 amino acids and a molecular weight of 59.2 kDa. Shorter variants of both receptor types are known. The main mode of action is regulation of gene expression. After agonist binding, the receptor forms a homodimer or heterodimer with another ER isoform and binds to specific estrogen responsive element in the promoter region of a target gene [22]. Beside the classic ligand-dependent nuclear action pathway of estrogen, there is a ligand-independent nuclear activation pathway that includes the phosphorylation of ER by a growth factor receptor. Furthermore, a third type of genomic responsive element-independent action is known [23]. The NO-dependent vasodilation induced by estrogen occurs in a matter of minutes. The onset is too rapid to be mediated by activation of mRNA and protein synthesis and is suggestive for a non-genomic signaling mechanism of ER [24,25]. Phosphorylation of eNOS at its Ser^1177^ activation site by the serine/threonine-kinase Akt and consequently increased NO release have been identified as the main signaling pathway. It is regulated by PI3K which lies further upstream and can be stimulated upon ER activation [26,27,28,29]. The rapid ER-induced eNOS activation is a membrane-bound process that involves the formation of a multi-molecular signaling complex. It is caveolin-1-dependent and occurs within the caveolae [30].

Human endothelial cells and vascular smooth muscle cells both express ERα and ERβ. ERα confers vascular protection and ERα splice variants are capable of mediating the cardiovascular favorable responses [30]. Human endothelial cells like human umbilical vein endothelial cells (HUVEC) express the full length ERα, ERβ and the *N*-truncated ER46 splice variant of ERα. However, the immortalized vascular endothelial cell line EA.hy 926 exclusively expresses ER46 and is unable to mediate the estrogen-dependent estrogen responsive elements transactivation but still retains the rapid signaling properties of estrogen [26]. The *N*-truncated ERα splice variant ER46 is preferentially found in endothelial caveolae fractions, especially in the presence of estrogen [31]. It is proposed that the ER46 variant is the predominant receptor in membrane-bound ER signaling and located at the center of the multi-molecular complex triggering the PI3K/Akt cascade and leading to eNOS activation [32].

Betulinic acid (BA, 3β-hydroxy-lup-20(29)-en-28-oic acid) is a pentacyclic triterpene of the lupane-type which is found in various plants. Its precursor molecule betulin is abundant in the bark of the white birch tree (*Betula alba*) [33]. Various biological properties of BA have been reported, including anti-tumor and anti-inflammatory effects. BA is an anti-tumor and anti-proliferative agent with a cytotoxic effect on numerous tumor cell lines like melanoma cell line or glioblastoma cells [34]. Probable molecular mechanisms are the modulation of NF-κB [35] and induction of the intrinsic mitochondrial apoptosis pathway [36]. BA and its semi-synthetic derivatives are potent antiviral agents that act as maturation inhibitors against the human immunodeficiency virus (HIV) [37]. The BA derivative bevirimat was tested in clinical phase I and phase II trials before being discontinued for efficacy reasons [38,39].

Beneficial cardiovascular effects of BA have been suggested. In a previous study, we have demonstrated that long-term incubation (18 h) of human endothelial cell line EA.hy 926 with BA increases eNOS expression and NO production through a PKC-independent mechanism [40]. A recently published study reported an increased CaMKII-dependent eNOS Ser^1177^ phosphorylation and increased NO production in EA.hy 926 cells upon stimulation with BA [41].

In the present study, we confirm that short term exposure to BA stimulates eNOS Ser^1177^ phosphorylation and NO formation in EA.hy 926 cells, and provide evidence for an ER- and PI3K/Akt-dependent signal transduction pathway.

## 2. Results

**Figure 1:**
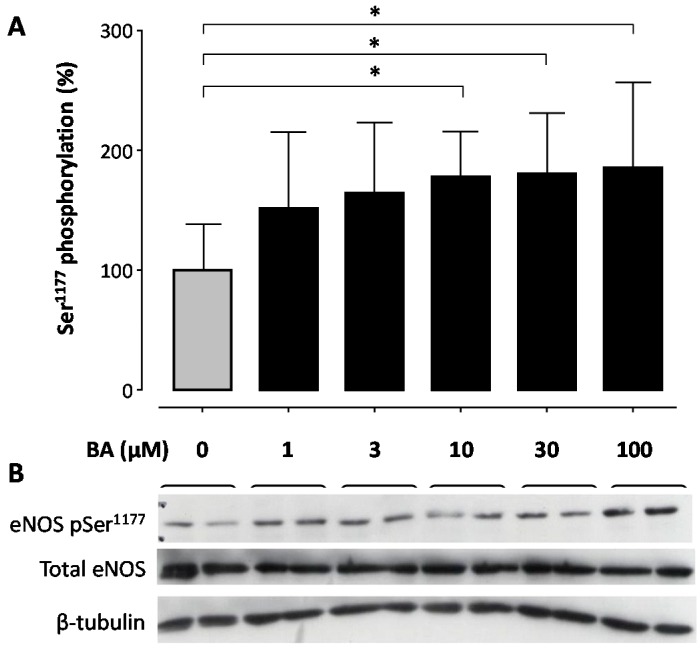
Betulinic acid (BA) increases phosphorylation of endothelial nitric oxide synthase (eNOS) at the Ser^1177^ residue in a concentration-dependent manner. Human EA.hy 926 endothelial cells were treated with 1, 3, 10 30 or 100 µM of BA for 30 min or the solvent control DMSO. (**A**) Western blot analysis was performed using a phospho-specific antibody and a total eNOS antibody. Total eNOS and β-tubulin were used for normalization. Columns represent arithmetic mean and standard deviation. * *p* < 0.05, compared to control using a one-way ANOVA followed by Dunnet’s multiple comparison test. *n* = 9; (**B**) shows representative blots.

**Figure 2:**
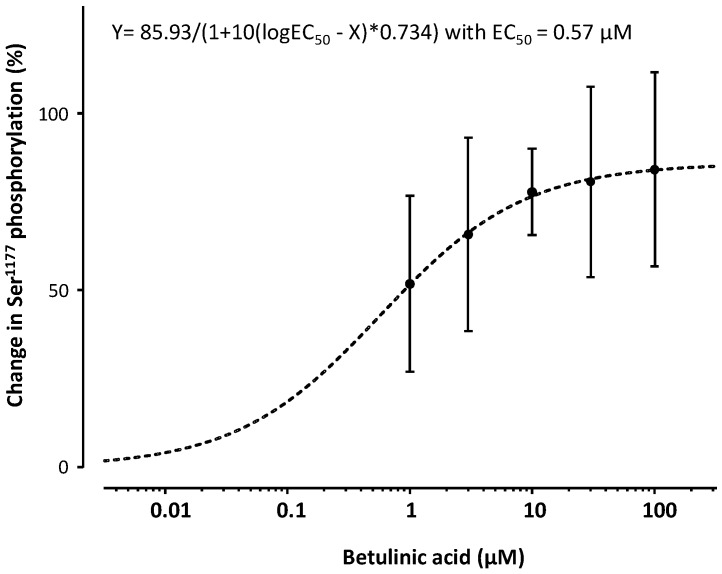
E_max_-Model of betulinic acid-induced percentual increase of eNOS phosphorylation at the Ser^1177^ residue. A Hill-equation with a maximum efficacy (E_max_) of 85.9 and a half-maximal effective concentration (EC_50_) of 0.57 µM and a shape factor of 0.7374 is the best fit for the experimental data.

**Figure 3:**
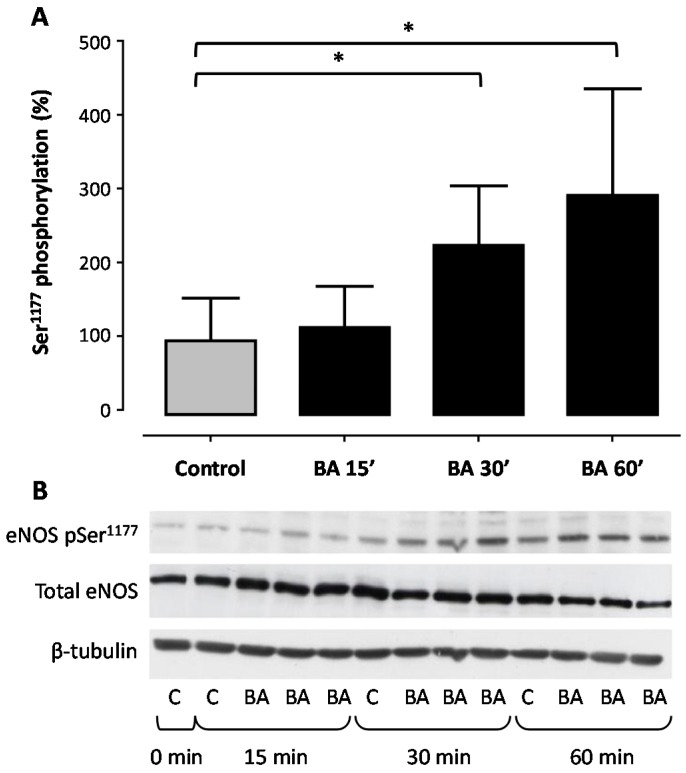
Betulinic acid (BA) increases eNOS phosphorylation at the Ser^1177^ residue of in a time-dependent manner. Human EA.hy 926 endothelial cells were treated with 30 µM BA for 15, 30 and 60 min. Controls (C) were incubated with 1‰ DMSO. (**A**) Western blot analysis was performed with a phospho-specific antibody and a total eNOS antibody. Total eNOS and β-tubulin were used for normalization. Columns represent arthmetic mean and standard deviation. * *p* < 0.05 compared to control using a one-way ANOVA followed by Dunnet’s multiple comparison test. *n* = 9; (**B**) shows representative blots.

Treatment of human EA.hy 926 endothelial cells with BA led to a concentration- (Figure 1 and Figure 2) and time-dependent (Figure 3) increase in eNOS phosphorylation at the Ser^1177^ residue. When incubated for 30 min, BA enhanced eNOS phosphorylation with a maximal increase of 85.93% (Figure 1) and a half-maximal effective concentration (EC_50_) of 0.57 µM (Figure 2).

**Figure 4:**
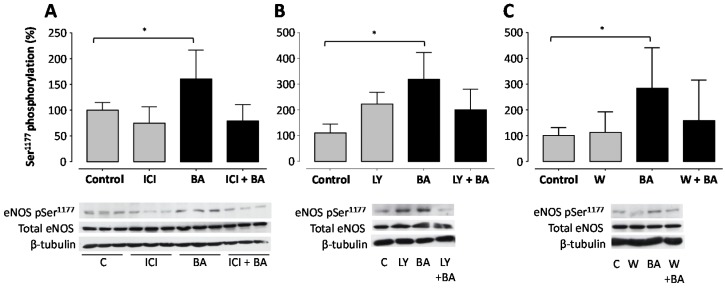
Betulinic acid (BA)-induced eNOS phosphorylation at the Ser^1177^ residue involves the estrogen receptor and the PI3K pathway. Human EA.hy 926 endothelial cells were pre-incubated for 60 min with the estrogen receptor antagonist ICI 182,780 (10 µM; panel (**A**)), or with the phosphoinositide 3-kinase (PI3K) inhibitors LY294002 (10 µM; panel (**B**)) and wortmannin (1 µM, panel (**C**)). Then, 30 µM BA were added to the cells and were incubated for another 60 min. Western blot analyses were performed with a phospho-specific eNOS-Ser^1177^ antibody and a total eNOS antibody. Total eNOS and β-tubulin were used for normalization. Columns represent arithmetic mean and standard deviation. * *p* < 0.05 compared to control using a one-way ANOVA followed by Dunnet’s multiple comparison test. *n* = 9.

Pre-treatment with the ER antagonist ICI 182,780 abolished BA-induced eNOS phosphorylation of the Ser^1177^ residue completely (Figure 4A). The eNOS phosphorylation level at the Ser^1177^ residue was slightly reduced when incubated with the anti-estrogen ICI 182,780 alone compared to the control group (statistically not significant). The PI3K inhibitors LY294002 and wortmannin both reduced BA-stimulated Ser^1177^ phosphorylation of eNOS (Figure 4B,C). Interestingly, both PI3K inhibitors only partially prevented BA-induced Ser^1177^ phosphorylation.

**Figure 5:**
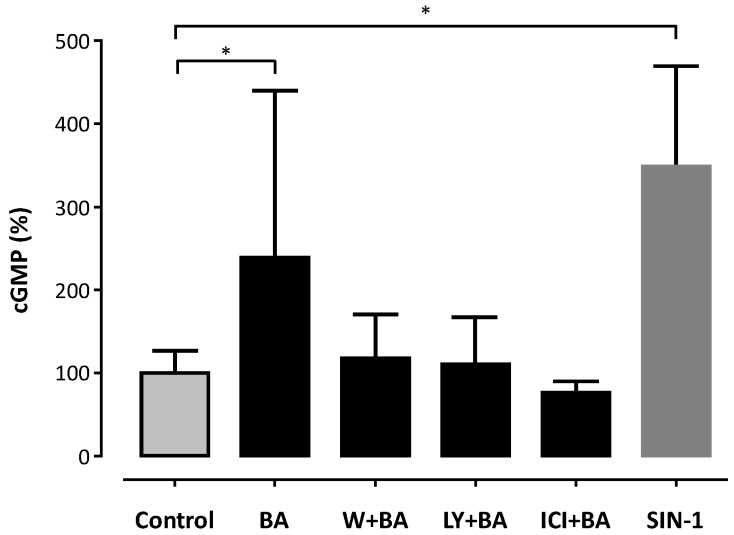
Betulinic acid (BA) stimulates nitric oxide (NO) production of EA.hy 926 cells. Human EA.hy 926 endothelial cells were pre-incubated with 1 µM wortmannin, 10 µM LY294002, 10 μM ICI 182,780 or 1‰ DMSO (controls) for 60 min. Then, 30 μM BA or DMSO was added and the cells were incubated for another 60 min. Bioactive NO from these cells was quantified using guanylyl cyclase-rich RFL-6 reporter cells. Positive controls were stimulated with SIN-1 for maximal cGMP production. The cyclic guanosine monophosphate (cGMP) content of the RFL-6 cells reflects NO production and was measured with a radioimmunoassay. Columns represent arithmetic mean and standard deviation. * *p* < 0.05 compared to control using a one-way ANOVA followed by Dunnet’s multiple comparison test. *n* = 9.

BA-induced enhancement of eNOS phosphorylation was associated with an increase in NO production, measured as cGMP content in RFL-6 reporter cells (Figure 5). An increase of 138% in cGMP content was observed upon incubation of EA.hy 926 cells with 30 µM BA for 60 min compared to baseline (Figure 5). Co-incubation with the ER antagonist ICI 182,780, or with the PI3K/Akt inhibitors wortmannin and LY294002 blocked the stimulatory effect of BA on NO production (Figure 5).

**Figure 6:**
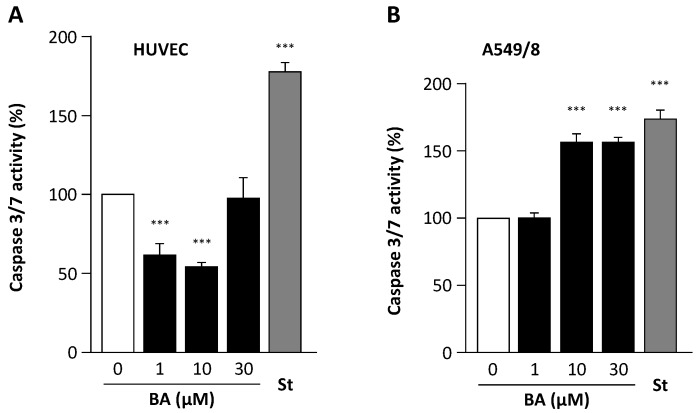
Effects of betulinic acid (BA) on apoptosis. Human umbilical vein endothelial cells (HUVEC, (**A**)) and alveolar epithelial carcinoma cell line A549/8 (**B**) were treated with BA, or staurosporine (St, 100 nM) as a positive control, for 18 h. The activity of caspase-3/7 was measured with the Caspase-Glo^®^ 3/7 luminescent assay (Promega Corporation, Madison, WI, USA). Columns represent mean and standard error of the mean. *** *p* < 0.001. *n* = 12.

BA has been shown to have proapoptotic effects in cancer cells. This effect could be confirmed in the alveolar epithelial carcinoma cell line A549/8 (Figure 6). Interestingly, BA did not induce apoptosis in HUVEC. At concentrations of 1 and 10 µM, betulinic acid even reduced caspase 3/7 activity in HUVEC (Figure 6).

## 3. Discussion

**Figure 7:**
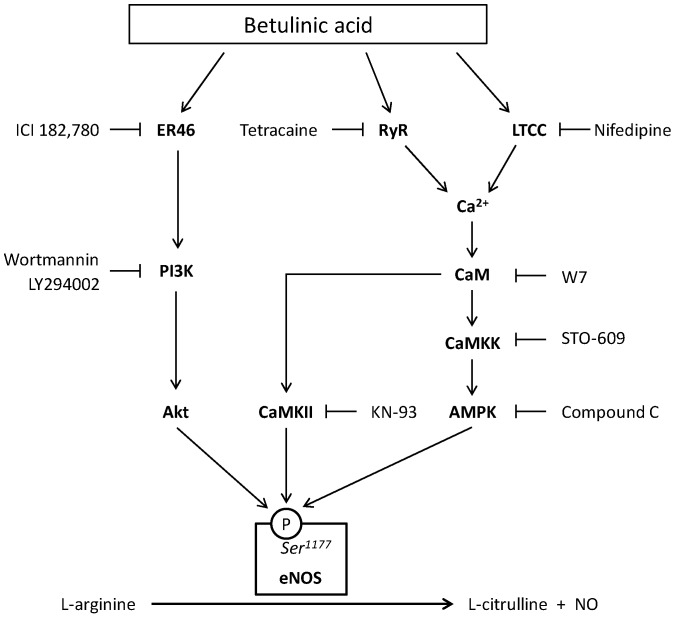
Proposed signaling pathways for betulinic acid (BA)-induced eNOS activation through phosphorylation of the Ser^1177^ residue in EA.hy 926 cells. BA-induced eNOS Ser^1177^ phosphorylation and NO formation are prevented by the estrogen receptor (ER) antagonist ICI 182,780 or by pharmacological inhibition of the phosphatidyl-inositol-3-kinase (PI3K)/Akt pathway with wortmannin or LY294002. In addition, a previous study [41] has demonstrated the involvement of l-type calcium channels (LTCC), ryanodine receptors (RyR), calmodulin (CaM), calcium calmodulin dependent kinase II (CaMKII), calcium calmodulin dependent protein kinase kinase (CaMKK), and AMP-activated protein kinase (AMPK).

In the present study, we demonstrate that BA stimulates eNOS Ser^1177^ phosphorylation and increases NO production in human endothelial cells. Both effects of BA can be reduced by ER blockade or by inhibition of the PI3K/Akt signaling pathway (Figure 7).

The short-term incubation of the immortalized human endothelial cell line EA.hy 926 with BA increases the production of bioactive NO (Figure 5). The fast onset is suggestive of an underlying post-translational regulation mechanism of eNOS activation rather than changes in eNOS expression. Phosphorylation of Ser^1177^ is the best studied of the regulatory sites known to increase eNOS activity [15]. Stimuli such as shear stress, insulin, bradykinin, estrogen and HMG-CoA reductase inhibitors (‘statins’) that activate eNOS lead to phosphorylation of Ser^1177^ [14,42,43]. The observed increase in bioactive NO production in our experiments correlates with the time- and concentration-dependent increase in the phosphorylation of the Ser^1177^ residue (Figure 1, Figure 2 and Figure 3).

Five distinct kinases have been reported to phosphorylate eNOS at Ser^1177^: PKA, PKG, Akt, AMPK and CaMKII [15]. These are counterbalanced by protein phosphatase 2A activity [44]. A calcium- and CaMKII/AMPK-dependent mechanism of BA-induced phosphorylation was recently proposed [41]. In the present study, we provide evidence that ER and Akt are involved in the signaling cascade of BA-induced Ser^1177^ phosphorylation. Treatment of cells with wortmannin and LY294002, inhibitors of PI3K, reduced the BA-induced Ser^1177^ phosphorylation (Figure 4). This indicates that the PI3K/Akt-signaling pathway mediates the eNOS activation through BA. The protein kinase Akt serves as a central hub for Ser^1177^ phosphorylation. It integrates various incoming signals from upstream pathways. Other naturally occurring products like epigallocatechin-3-epigallate have similar eNOS-activating effects mediated through PI3K/Akt-dependent signaling [45]. Interestingly, blockade of PI3K did not fully inhibit the BA-induced Ser^1177^ phosphorylation (Figure 4); a similar observation was made in 1999 by Fulton et al. for other agonists activating eNOS through PI3K/Akt, implicating that parallel pathways might be involved [46]. 

Both receptor-dependent and -independent agonists can activate eNOS as a consequence of an increase in the intracellular concentration of free calcium and association of the calcium/CaM complex with the enzyme [42]. NO production can also be enhanced in the absence of a maintained increase in intracellular calcium. eNOS Ser^1177^ phosphorylation is, however, only associated with a modest ~2-fold increase in enzyme activity. It cannot account for the burst of NO production observed after the activation of endothelial cells by calcium-elevating agonists [42].

The mechanism proposed by Jin et al. comprises BA-stimulated increase in intracellular free calcium levels via extracellular calcium entry through l-type calcium channels and calcium release from ryanodine receptors (Figure 7). The increase in intracellular calcium concentrations causes phosphorylation of CaMKII, CaMKK, and its downstream kinase AMPK, activating eNOS und increasing NO production through Ser^1177^ phosphorylation. The BA-induced eNOS Ser^1177^ phosphorylation was attenuated by the CaM antagonist W7 and the CaMKII inhibitor KN-93 as well as the AMPK inhibitor STO-609 [41]. Still, inhibition of CaMKII and AMPK did not totally abolish the increase in Ser^1177^ phosphorylation compared to baseline. Also ryanodine receptor inhibition with tetracaine and l-type calcium channel inhibition with nifedipine did not fully block Ser^1177^ phosphorylation [41]. We therefore postulate that BA-induced eNOS activation is dependent on both calcium-mediated signaling, and PI3K/Akt-mediated signaling. 

The anti-estrogen ICI 182,780 completely blocked the BA-induced eNOS phosphorylation of Ser^1177^ and the BA-induced increase in production of bioactive NO (Figure 5). This is suggestive of involvement of ER in the BA-induced eNOS activation. Our findings are consistent with the fact that estrogen itself is an eNOS activator that perpetuates its signal through the PI3K/Akt pathway and ultimately leads to Ser^1177^ phosphorylation [28]. ER has been shown to form a multi-molecule membrane-bound complex that activates PI3K and subsequently Akt to finally phosphorylate eNOS at Ser^1177^ [28,30,47]. ICI 182,780 is an anti-estrogen developed as a second line drug in breast cancer therapy. It acts as a pure antagonist on ERα and ERβ receptors with no agonist effect [48]. Our findings are a strong argument for the involvement of ER in the signaling of BA-induced eNOS phosphorylation at Ser^1177^. 

Human endothelial cells like HUVEC express both ERα and ERβ [49]. ER-mediated eNOS activation is only mediated by the α-isoform [50]. The endothelial cell line we used in our experiments, EA.hy 926, neither express ERβ nor the normal 66 kDa variant of ERα but its *N*-truncated 46 kD splice variant, ER46. ER46 lacks the ability of transactivating genomic effects but retains rapid non-genomic signaling capacities. It mediates the estrogen-stimulated eNOS activation in the EA.hy 926 cell line [32]. Because BA induces an eNOS-phosphorylation at Ser^1177^ via the PI3K/Akt pathway and an anti-estrogen blocks the signaling cascade initiated by treatment with BA, an activation of ER46 splice variant similarly to estrogen and subsequently a signalosome involving PI3K and Akt by BA is conceivable. We conclude that BA stimulates eNOS phosphorylation and NO production by activation of an ER46-mediated, PI3K/Akt- and CaMKII/AMPK-dependent pathway. This kind of dual pathway signaling has been described previously for eNOS activation induced by the isoflavone puerarin [51].

Various beneficial effects of BA on endothelial cell function, cardiovascular health and cerebrovascular health have been reported so far. On a short term scale, NO production is increased through post-translational activation. On a longer term scale, BA is capable of increasing the expression of eNOS and reducing the expression of NADPH oxidase as shown in our previous study [40]. Down-regulation of NADPH oxidase reduces oxidative stress, which in turn not only prevents NO inactivation by superoxide (and thus enhances NO bioavailability) but also prevents eNOS uncoupling (and thus enhances NO production) [40]. In addition, we also observed an anti-apoptotic effect of BA in endothelial cells (Figure 6). The protection of endothelial cells from apoptosis by BA may represent a mechanism contributing to the previously reported protective effect of BA against cerebral ischemia-reperfusion injury in mice [52]. Another property with an impact on cardiovascular health is the recently reported prevention of abdominal fat accumulation in mice fed with a high-fat diet [53]. This makes BA an interesting molecule for possible treatment or prevention of cardiovascular disease. Despite the demonstrated beneficial effects of BA by several mechanisms, the low solubility and poor permeability hinders the further development of this promising novel agent. BA is orally active but due to poor bioavailability, large doses are required [54]. Further investigations should focus on the ability of BA derivatives with a more favorable pharmacokinetic profile that are orally bioavailable to humans, such as bevirimat, to increase endothelial NO production. Concerning the ER-dependent signaling mechanism, further investigations to elucidate BAs binding affinity and selectivity for the different ER isoforms and impact on downstream genomic and non-genomic signaling are necessary.

ER isoforms are widely expressed throughout different organ systems and ER signaling is involved in various disease such as breast cancer, or arteriosclerosis, and physiological processes such as metabolic homeostasis, and skeletal maturation [55]. From a clinical point of view, the beneficial and potentially detrimental health effects of BA-mediated modulation of estrogen signaling need to be addressed. Potential applications, where ER has been investigated as therapeutic target, are conceivable, e.g., to alleviate post-menopausal symptoms, reduce the risk for cardiovascular disease, as well as in diseases affecting the CNS such as depression, Alzheimer’s disease, and Parkinson’s disease [55]. Detrimental effects such as drug-interactions with anti-estrogens during therapy of hormone receptor positive breast cancer have to be answered, especially because BA-containing formulations are available over-the-counter as dietary supplements.

In summary, our study confirms that BA increases eNOS activity in endothelial cells. The effect can be attenuated by PI3K inhibitors and can be blocked by the ER antagonist ICI 182,780. We postulate that fast non-genomic effects of the ER46 splice variant with downstream activation of PI3K/Akt and consecutive eNOS phosphorylation at Ser^1177^ are involved in BA-induced eNOS activation.

## 4. Materials and Methods

### 4.1. Chemicals

^125^I-cGMP with a specific activity of 81.4 TBq/mmol was obtained from Biotrend (Cologne, Germany). 2-Mercaptoethanol, 4-(2-hydroxyethyl)-1-piperazineethanesulfonic acid (HEPES), acetic acid, ethanol, glucose, isopropyl alcohol, milk powder, methanol, *N*,*N*,*N*’,*N*’-tetramethylethylene-diamine (TEMED), sodium acetate, sodium dodecyl sulfate (SDS), Tris-buffer, and Tween^®^ 20 were obtained from Roth (Karlsruhe, Germany). 3-Isobutyl-1-methylxanthine (IBMX), 3-morpholino-sydnonimine (SIN-1), ammonium persulfate (APS), betulinic acid (BA), bicinchoninic acid, bromophenol blue, copper sulfate, gamma-globulin, *n*-butanol, sepiapterin, sodium fluoride, sodium pyrophosphate, and Triton-X 100 were obtained from Sigma (Taufkirchen, Germany). Acrylamide, 30% solution (37.5:1), and bovine serum albumin (BSA), albumin fraction V were obtained from Applichem (Darmstadt, Germany). ICI 182,780 was obtained from Tocris Bioscience (Minneapolis, MN, USA). LY294002, and wortmannin were obtained from Calbiochem (Darmstadt, Germany).

### 4.2 Cell Lines

EA.hy 926: A human endothelial cell line resulting from a fusion of human umbilical vein endothelial cells and A549/8 (human alveolar epithelium-like lung carcinoma cells) and that expresses eNOS. The cells were kindly provided by Cora-Jean S. Edgell, University of North Carolina at Chapel Hill, USA. RFL-6: Fibroblasts from fetal rat lungs that express soluble guanylyl cyclase but do not express eNOS. The cells (CCL-192) were obtained from ATCC (Manassas, VA, USA).

### 4.3. Cell Culture

EA.hy 926 endothelial cells [56] were seeded on culture dishes or 6-well-plates containing Dulbecco’s Modified Eagle Medium (Thermo Scientific, Waltham, MA, USA) and 10% fetal calf serum supplemented with l-glutamine (2 mM), penicillin (100 UI/mL), streptomycin (100 µg/mL), sodium pyruvate (1 mM) and 1xHAT (hypoxanthine, aminopterin, thymidine) (Invitrogen/Thermo Fisher Scientific, Waltham, MA, USA) [57] and cultured at 5% CO_2_. Cells were used between passage 6 and 14. RFL-6 cells were seeded on 6-well-plates containing Ham’s F12 medium (Thermo Scientific, Waltham, MA, USA) and 15% FCS supplemented with penicillin (100 IU/mL) and streptomycin (100 µg/mL) and cultured at 5% CO_2_. Cells were used between passage 6 and 8 as reporter cells for the determination of NO production by EA.hy 926 cells [58].

### 4.4. Western Blots

Confluent EA.hy 926 cells were incubated with ascending concentrations of BA for time periods of 15, 30 and 60 min. In case of experiments with the PI3K/Akt or ER inhibitors the cells were pre-incubated for 1 h with the inhibitor prior to the administration of BA. In all experiments, BA and the inhibitors were dissolved in DMSO. Stock solutions and aliquots were kept at −80 °C. An equal volume of DMSO without BA or the inhibitor was administered as control. Cells were lysed with Tris-HCl buffer (20 mM) containing NaCl (137 mM), sodium pyrophosphate (50 mM), NaF (50 mM), Na_2_P_2_O_7_ (200 µM), 1% Triton X-100, HALTTM Protease and Phosphatase Inhibitor Single-use Cocktail #78442 (Thermo Scientific) left on ice for 10 min and then centrifuged at 13,000 rpm for 15 min. 15 µg of the protein in the cell supernatant were heated with SDS-PAGE sample buffer, separated by SDS-polyacrylamide gel electrophoresis (PAGE) and electroblotted onto nitrocellulose membrane (Schleicher and Schuell, Düren, Germany). Prestained molecular weight marker proteins (#SM0671, Fermentas, Burlington, ON, Canada) were used. The blots were cut vertically at the height of the 77 kDa-marker. After washing the upper part for 1 h at room temperature in a buffer containing Tris (20 mM, pH 7.4), 5% dryed milk powder and 0.1% Triton X-100, the blots were incubated with mouse monoclonal anti-eNOS antibody (1:1000) (BD Bioscience, San Jose, CA, USA) or rabbit phosphor-specific anti-Ser^1177^ eNOS antibody (1:2000) (Cell Signalling Technology, Danvers, MA, USA) at 4 °C for 16 h in a buffer containing Tris (20 mM, pH 7.4), NaCl (137 mM), 3% bovine serum albumin and 0.1% Triton X-100. The lower part was washed for 1 h at room temperature in a buffer containing Tris (20 mM, pH 7.4), 3% bovine serum albumin and 0.1% Triton X-100 before incubation with mouse β-tubulin antibody (1:200,000) (Sigma Aldrich, St. Louis, MO, USA) at 4 °C for 16 h in a buffer containing Tris (20 mM, pH 7.4), NaCl (137 mM), 5% dried milk powder and 0.1% Triton X-100. After a subsequent washing the blots were incubated with peroxidase-conjugated anti-mouse or anti-rabbit antibody at room temperature for 1 h, washed again and visualized using a commercially available enhanced chemiluminescence kit (ECL Kit, PerkinElmer, Waltham, MA, USA) according to the manufacturer’s instruction [58].

### 4.5. NO Measurement through cGMP-Reporter Cell Assay

To determine NO production from EA.hy 926 cells, a bioassay with RFL-6 rat lung fibroblasts as reporter cells was used [59]. Confluent EA.hy 926 cells were incubated for 1 h with 30 µM BA in Locke’s solution (154 mM NaCl, 5.6 mM KCl, 2 mM CaCl_2_, 1 mM MgCl_2_, 3.6 mM NaHCO_3_, 5.6 mM d-Glucose, 10.0 mM HEPES, pH 7.4) containing 50 µM L-arginine and 3 µM sepiapterin. In case of experiments with inhibitors, the cells were pre-incubated with the inhibitor for 1 h prior to the administration of BA. The RFL-6 cells were pre-incubated for 30 min with Locke’s solution containing 600 µM IBMX. After the treatment with BA, EA.hy 926 were washed with Locke’s solution and incubated for 3 min at 37 °C in Locke’s solution containing 200 U/mL SOD, 600 µM IBMX, 50 µM l-arginine, 3 µM sepiapterin and 30 µM BA. The media containing the NO released by the EA.hy 926 cells were transferred onto the guanylyl cyclase-rich RFL-6 cells for another 3 min of incubation. To stop the reaction, the medium was removed, 1 mL of ice cold 50 mM sodium acetate solution (pH 4.0) was added to the wells and the cells were rapidly frozen with liquid nitrogen. A radioimmunoassay was performed to determine the cGMP content of the RFL-6 cells as described previously [60]. The basal cGMP content of the RFL-6 cells was substracted from all samples. 

### 4.6. Statistics

Results are given as arithmetic mean value ± standard deviation. Statistical differences between mean values were determined by analysis of variance (ANOVA) followed by Dunnet’s multiple comparison test using Prism 6.02 (GraphPad Inc., La Jolla, CA, USA). Fitting of experimental data to a Hill-equation was performed using Prism 6.02 (GraphPad).

## 5. Conclusions

Our study shows that betulinic acid stimulates eNOS activity in endothelial cells. The signaling cascade involves the estrogen receptor variant ER46 and the PI3K/Akt kinase pathway.
